# Drug-target interaction prediction with tree-ensemble learning and output space reconstruction

**DOI:** 10.1186/s12859-020-3379-z

**Published:** 2020-02-07

**Authors:** Konstantinos Pliakos, Celine Vens

**Affiliations:** 1 KU Leuven, Campus KULAK, Faculty of Medicine, Kortrijk, Belgium; 2 ITEC, imec research group at KU Leuven, Kortrijk, Belgium

**Keywords:** Drug-target networks, Network reconstruction, Interaction prediction, Tree-ensembles, multi-output prediction

## Abstract

**Background:**

Computational prediction of drug-target interactions (DTI) is vital for drug discovery. The experimental identification of interactions between drugs and target proteins is very onerous. Modern technologies have mitigated the problem, leveraging the development of new drugs. However, drug development remains extremely expensive and time consuming. Therefore, in silico DTI predictions based on machine learning can alleviate the burdensome task of drug development. Many machine learning approaches have been proposed over the years for DTI prediction. Nevertheless, prediction accuracy and efficiency are persisting problems that still need to be tackled. Here, we propose a new learning method which addresses DTI prediction as a multi-output prediction task by learning ensembles of multi-output bi-clustering trees (eBICT) on reconstructed networks. In our setting, the nodes of a DTI network (drugs and proteins) are represented by features (background information). The interactions between the nodes of a DTI network are modeled as an interaction matrix and compose the output space in our problem. The proposed approach integrates background information from both drug and target protein spaces into the same global network framework.

**Results:**

We performed an empirical evaluation, comparing the proposed approach to state of the art DTI prediction methods and demonstrated the effectiveness of the proposed approach in different prediction settings. For evaluation purposes, we used several benchmark datasets that represent drug-protein networks. We show that output space reconstruction can boost the predictive performance of tree-ensemble learning methods, yielding more accurate DTI predictions.

**Conclusions:**

We proposed a new DTI prediction method where bi-clustering trees are built on reconstructed networks. Building tree-ensemble learning models with output space reconstruction leads to superior prediction results, while preserving the advantages of tree-ensembles, such as scalability, interpretability and inductive setting.

## Background

Predicting accurately drug-target interactions (DTI) is vital for the development of new drugs. Accurate and efficient identification of interactions between drugs and target proteins can accelerate the drug development process and reduce the required cost. In addition, the identification of drug-target interactions can unveil hidden drug or protein functions and shed light to enigmatic disease pathology mechanisms [1]. It can also provide scientists with insights which help in foreseeing adverse effects of drugs [2, 3]. Furthermore, apart from discovering new drugs, DTI prediction can also leverage drug repositioning [2, 4–6], which aims at revealing new uses for already approved drugs. However, despite the persisting efforts made by the scientific community, experimentally identifying DTIs remains extremely demanding in terms of both time and expenses [7, 8]. The employment of computational methods and especially machine learning for in silico DTI prediction is thereby crucial for drug discovery and repositioning. Machine learning models can direct experiments, reveal latent patterns in large scale drug or protein data collections and extract unprecedented knowledge in drug-target networks.

Machine learning has shown great potential when employed in medicine and bioinformatics, especially in prediction or clustering tasks [9–11]. The most appealing field of machine learning is the supervised learning, where the learning models are constructed on an input set $\mathcal {X}$ and an output set $\mathcal {Y}, (f:\mathcal {X} \rightarrow \mathcal {Y})$. The instances (e.g., drugs, proteins) are represented by a set of feature vectors and they are also associated with an output variable. The goal is the learning of a function, based on the features of a training set of instances, which predicts the output [12]. In inductive modelling, when this function (model) is built, one can employ it to predict the output of new instances. The task is called regression in cases where the output is numeric and classification when it is categorical.

### Multi-output prediction in drug discovery

An interesting extension of typical classification or regression problems is the task of multi-output (multi-target) prediction [13]. In this case, the model learns to predict multiple output variables at the same time. Subcategories of multi-target prediction are multi-target classification (i.e., the targets have categorical values) and multi-target regression [14]. A distinctive condition is multi-label classification [15, 16]. This can be translated as multi-target regression with only zero and one as numeric values for each target, or as multi-target classification, with only binary values for each target.

Multi-output prediction models learn from multiple outputs simultaneously. They are often benefited from exploiting possible correlations between the targets, improving this way their prediction performance. In particular, when it comes to drug discovery, the interest in multi-output models is even greater. In the past, the learning methods proposed for DTI prediction aimed at performing predictions for a specific target protein, admitting the old paradigm of ‘*one target, one drug, one disease*’. This strategy led to inferior performance as the drug-disease relation complexity is far greater [17, 18]. The majority of known diseases are usually associated with multiple proteins [19]. It has been generally admitted that drugs which interact with multiple target proteins (*polypharmacology*) are more effective [20–22]. Multi-output learning can also contribute to investigating the off-target drug activity (i.e., unintended function of a drug). The investigation of such activities can lead to new uses for existing drugs (drug repositioning) or contrarily, the identification of unwanted side-effects. Such adverse reactions of drug candidates are usually identified at a later stage of the drug development process, leading to extremely expensive late stage failures.

### DTI networks

**Fig. 1:**
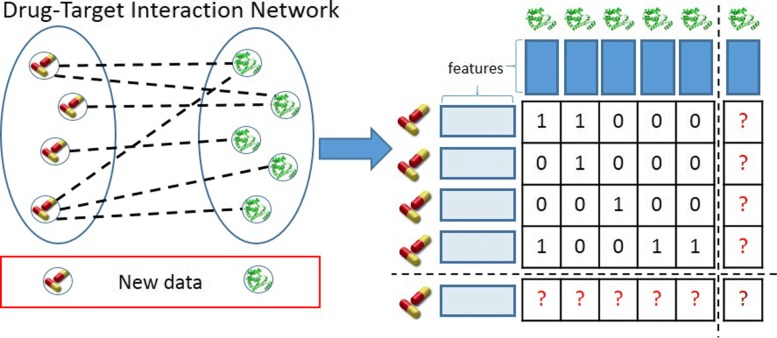
Illustration of a (bi-partite) DPI interaction network

A drug-protein interaction network is a heterogeneous network (also referred to as bi-partite graph) that can be formulated as a collection of two sets of items that interact with each other. Each item set is described by its own features which compose the background information in our problem. The interactions are the links connecting the nodes of the network and are often represented as a matrix, often denoted as interaction, adjacency, or connectivity matrix. In this paper, we use the term interaction matrix. In Fig. 1, an illustration of a DTI network in the aforementioned setting is displayed. One can follow two learning strategies in this framework: the local [23] and the global [24]. A discussion of these two strategies took place originally in [25] and later in [26, 27].

Traditional DTI prediction models based on the local approach handle the two sets of the DTI network separately. In particular, they first divide the DTI network into different (traditional) feature sets, the drug-based set and the protein-based one. Next, each set’s learning task is tackled separately and then the results are combined. Often, in the absence of information on both sides, local models are built on a single feature space, ligand (drug) space or target protein space. Ligand-based models are built on the known ligands that interact with the target proteins. However, the performance of these models is impaired when it comes to target proteins with only a really small number (or even none) of known binding ligands [28]. Alternatively, target-based models are built on the target proteins using protein (3*D*) structure information. Nevertheless, the 3*D* structure of many target proteins is often unavailable.

Due to these bottlenecks, the interest of the scientific community was shifted towards a global setting referred to as *chemogenomics* [29, 30]. The underlying idea behind the global setting is that drug information is integrated with target protein information and thereby complement each other. However, this setting also suffers from weaknesses. Global approaches are mostly based on matrix factorization or graph learning, following the transductive setup (i.e., the test instances are needed in the training phase). Alternatively, there are other approaches which are based on inductive classifiers. In these cases, DTI prediction is treated as a binary classification problem where classifiers are trained over the Cartesian product of drug-related and target-related feature sets. This Cartesian product often leads to an enormous data matrix. Thus, these approaches are computationally very expensive and not particularly scalable. Furthermore, in this global setting, one assumes that rich background information (feature vectors) is always available for both all drugs and all their targets, which is not always the case. Despite these disadvantages, global approaches remain the most promising.

### Introduction to the proposed method

Major problems in DTI prediction are the present noise in the output space, the existence of no true negative interactions and the extreme class imbalance. These problems are not easily surpassed and they often devastate the predictive performance of even powerful learning methods. There is a plethora of studies aiming at feature space transformation, removing noise or revealing latent manifolds in the data. However, to the best of our knowledge, there is almost nothing on integrating supervised learning methods with output space reconstruction. An intelligent reconstruction can remove the existing noise, reveal latent patterns and mitigate class imbalance in the output space.

In this paper, we propose a new DTI prediction framework that provides great predictive performance while being computationally efficient and scalable. We propose that building multi-output learning models on reconstructed networks leads to superior predictive performance. Our approach addresses DTI prediction as a multi-output prediction task, building tree-ensemble learning models and specifically ensembles of bi-clustering trees (eBICT) [27, 31], on reconstructed networks. Although other inductive learning models could have been employed, we designate eBICT because it inherits the merits of tree-ensembles, such as scalability, computational efficiency, and interpretability. eBICT also provides bi-clustering [32] of the interaction matrix as a side product.

Reconstructing a DTI network is a challenging problem and various approaches have been proposed over the years. The most effective approaches are typically related to matrix factorization. Scientists have extended the traditional optimization problem of matrix factorization including multiple constraints. Recently, a neighborhood regularized logistic matrix factorization (*NRLMF*) [33] method was presented, integrating logistic matrix factorization (LMF) with neighborhood regularization taking also into account class imbalance. The authors obtained outstanding results, naming their method a state of the art in DTI prediction. Here, we employ NRLMF for reconstructing the target space in our problem and we show that the predictive performance of inductive learning models is particularly boosted when they are integrated with output space reconstruction. The proposed multi-output prediction framework combines great prediction performance with scalability, computational efficiency, and interpretability. The proposed method offers bi-clustering of a drug-target network as a side product and also follows the inductive setup. The latter means that neither the test instances are needed in the training process nor the training instances are required to perform predictions for new instances. Furthermore, the proposed method is apt to perform predictions for new candidate drugs, a setting applied to drug discovery, new target proteins, a setting more applied to drug repositioning, or new drug-protein pairs.

### Related work

Recently, great interest has been witnessed in developing machine learning models for DTI prediction [34]. Kernel learning was employed for DTI prediction in [35], where the authors constructed kernels for drugs, target proteins and the interaction matrix. DTI prediction was then performed using the regularized least squares classifier. This approach was later extended to handle new candidate drugs or target proteins in [36]. In [37], a semi-supervised approach was proposed integrating similarities between drugs and local correlations between targets into a robust PCA model. Deep learning strategies for DTI prediction were used in [38, 39]. An interesting multi-label classification framework exploiting label partitioning was recently proposed for DTI prediction in [40] as well as in the 7th chapter of [41]. Furthermore, the authors in [42] employed multi-domain manifold learning and semidefinite programming for DTI prediction while in [43] it was handled using label propagation with linear neighborhood information. Moreover, Shi et al. [44] presented an MLkNN [45] driven approach to predict interactions between new candidate drugs and target proteins. The method was based on clustering the features of the target proteins. A second interaction matrix was constructed based on this super-target clustering. The MLkNN was applied to both interaction matrices and final predictions were yielded as an integration of the individual prediction scores. MLkNN was also used in [46] for drug side effect prediction. A feature selection-based MLkNN method was presented, which combined the construction of multi-label prediction models with the determination of optimal dimensions for drug-related feature vectors.

Many promising predictors were based on matrix factorization [30]. For instance, in [47], graph regularization was incorporated into matrix factorization. In particular, the proposed method consisted of two steps. First, a weighted k Nearest Neighbor (k-NN) was employed, converting the binary interaction scores into numeric ones. Next, a graph regularization driven matrix factorization method was applied. In [33], the authors proposed a neighborhood regularized logistic matrix factorization (NRLMF) approach. Their method incorporated neighborhood regularization into logistic matrix factorization. The performance of their approach was also enhanced by applying a weighing scheme that favored the pairs where an interaction occurs. In [29], another similar extension to logistic matrix factorization (LMF) was presented. The authors integrated LMF with multiple kernel learning and graph Laplacian regularization.

Extensive work has been also noted in building ensemble learning models. In more detail, a synergistic model was built in [28]. It achieved a fair predictive performance integrating predictions from multiple methods into a Learning to Rank framework. In [48], ensemble learning was also used along with strategies tackling existing class-imbalance in drug-target networks.

Moreover, several approaches emphasized on transforming or extending the feature space, generating more informative representations of the DTI network. Next, the final predictions were yielded as the output of a common classifier. In [49], the authors used network (graph) mining to extract features. Next, a Random Forest (RF) [50] classifier was applied to predict the interactions. Similarly in [51], the authors exploited the topology of the DTI network to extract features. The final predictions were performed using a Random Forest classifier. In addition, Liu et al. [52] proposed a strategy to identify highly negative samples before applying a classifier.

## Results

### Evaluation metrics

In order to evaluate the proposed approach we employed two metrics in a micro-average setup, namely area under the receiver operating characteristic curve (AUROC) and area under precision recall curve (AUPR). ROC curves correspond to the true positive rate $\left (\frac {TP}{TP+FN}\right)$ against the false positive rate $\left (\frac {FP}{FP+TN}\right)$ at various thresholds. Precision-Recall curves correspond to the Precision $\left (\!\frac {TP}{TP+FP}\!\right)$ against the Recall $\left (\!\frac {TP}{TP+FN}\!\right)$ at various thresholds.

In Table 3 it can be seen that the interaction datasets are very sparse, which makes the corresponding classification task very class imbalanced. Generally, AUPR is considered more informative than AUROC in highly imbalanced classification problems [53, 54]. Nevertheless, it is important to note that in drug discovery the crucial value is to minimize the false negatives (FN), these are interactions which are positive but overlooked by the computational predictor. Any positive in silico predictions will get validated in the lab, whereas strong negative ones are rarely checked.

### Evaluation protocol

A major point in our paper is to evaluate the contribution of output space reconstruction to the predictive performance of multi-output learning models. To this end, our evaluation study begins with comparing the proposed DTI approach (BICTR) against ensemble of bi-clustering trees (eBICT) without output space reconstruction. Next, we compare BICTR to three state of the art DTI prediction methods, BLMNII [36], STC [44], and NRLMF [33]. The method in [36] is denoted as BLMNII and is a kernel-based local approach. The method in [44] is denoted as super target clustering (STC). It uses MLkNN in a target clustering-driven strategy. The methods are compared in the three prediction settings presented in the “Method” section, namely *T*_*d*_×*L*_*p*_,*L*_*d*_×*T*_*p*_, and *T*_*d*_×*T*_*p*_. We performed comparisons independently for every setting. Both BLMNII and STC are local models and the predictions between pairs of new drugs and new targets were performed following the standard two step approach proposed in [26, 55].

In *T*_*d*_×*L*_*p*_ and *L*_*d*_×*T*_*p*_ we used 10-fold cross validation (*CV*) on nodes (i.e., CV on drugs and CV on targets, respectively). It is important to clarify that when a drug *d*_*i*_ is included in the test set of the *T*_*d*_×*L*_*p*_ setting the whole interaction profile of *d*_*i*_ should not be present in the training set. The same holds for the target proteins in the *L*_*d*_×*T*_*p*_ setting. In *T*_*d*_×*T*_*p*_, we used *CV* on blocks of drugs and targets. For every iteration, we removed one fold corresponding to drugs and one fold corresponding to proteins from the learning set and used their combined interactions as test set. When a drug-target pair (*d*_*i*_,*p*_*j*_) is included in the test set this means that the whole interaction profile of both *d*_*i*_ and *p*_*j*_ should not be present in the training set. In *T*_*d*_×*T*_*p*_, we used 5-fold *CV* over blocks of drugs and targets (i.e., 5×5=25 folds). This was done because the data are very sparse and the application of a 10-fold *CV* setting was difficult.

The number of trees in tree-ensemble algorithms was set to 100 without tree-pruning. The parameter *c* in Eq.2, which defines the weight of the positive (interacting) drug-target pairs, was set equal to 5 as in [33]. All the other parameters of NRLMF, shown in Eq. 2, were optimized in a 5-fold CV inner tuning process (nested CV) following grid search. More specifically, parameters *λ*_*d*_,*λ*_*p*_,*α*,*β* as well as the optimal learning rate were selected from a range of {2^−2^,2^−1^,2^0^,2^1^}. The number of nearest neighbors was selected from {3,5,10} and the number of latent factors from {50,100}. For BLMNII, we used the rbf kernel as proposed in the corresponding paper and tuned the linear combination weight through 5-fold CV inner tuning (nested CV), picking values in {0.1,0.25,0.5,0.75,1.0,1.25,1.5}. The number of nearest neighbors in STC was also tuned through 5-fold CV inner tuning (nested CV), picking values in {3,5,7,9,11}.

### Obtained results

The AUROC and AUPR results are presented in Tables 1 and 2, respectively. Best results are shown in bold faces and * indicates that the results between BICTR and its competitor were found statistically significantly different (*p*<0.05) based on a Wilcoxon Signed-Ranks Test run on the CV-folds. As it is reflected, BICTR outperforms eBICT in all three prediction settings, in terms of both AUROC and AUPR. Specifically, BICTR significantly outperforms eBICT in every dataset in terms of AUROC. It also achieves better AUPR results in every dataset and setting. The only exceptions occur in the E dataset in *T*_*d*_×*L*_*p*_ and *T*_*d*_×*T*_*p*_ where nonetheless the differences are not statistically significant. Thus, the original hypothesis that network reconstruction can boost the predictive performance of multi-output learning models is verified.

**Table 1:** AUROC results for the compared methods

AUROC					
Data	*BICTR*	*eBICT*	*NRLMF*	*BLMNII*	*STC*
	*T*_*d*_×*L*_*p*_>				
NR	**0.875**	0.787*	0.851*	0.807*	0.794*
GR	**0.894**	0.857*	0.867*	0.842*	0.847*
IC	**0.811**	0.780*	0.792	0.737*	0.783*
E	**0.891**	0.827*	0.777*	0.815*	0.794*
Avg	**0.868**	0.813	0.822	0.800	0.805
	*L*_*d*_×*T*_*p*_				
NR	**0.905**	0.614*	0.747*	0.667*	0.525*
GR	**0.951**	0.846*	0.861*	0.776*	0.800*
IC	**0.968**	0.931*	0.949*	0.887*	0.909*
E	**0.973**	0.924*	0.940*	0.904*	0.906*
Avg	**0.949**	0.829	0.874	0.809	0.785
	*T*_*d*_×*T*_*p*_				
NR	0.676	0.634*	0.683	0.554*	0.469*
GR	**0.811**	0.792*	0.800*	0.475*	0.630*
IC	**0.733**	0.719*	0.731	0.466*	0.649*
E	**0.812**	0.785*	0.749*	0.490*	0.682*
Avg	**0.758**	0.733	0.741	0.496	0.608

**Table 2:** AUPR results for the compared methods

AUPR					
Data	*BICTR*	*eBICT*	*NRLMF*	*BLMNII*	*STC*
	*T*_*d*_×*L*_*p*_				
NR	0.480	0.444*	**0.501**	0.436	0.467
GR	0.334	0.329	**0.349**	0.312	0.324
IC	**0.329**	0.317*	**0.329**	0.213*	0.307
E	0.314	0.316	0.367*	0.255*	0.353
Avg	0.364	0.352	**0.387**	0.304	0.363
	*L*_*d*_×*T*_*p*_				
NR	**0.555**	0.424*	0.485*	0.338*	0.384*
GR	**0.523**	0.504*	0.406*	0.324*	0.365*
IC	**0.808**	0.791*	0.798	0.724*	0.779*
E	0.785	0.784	0.795*	0.735*	0.752*
Avg	**0.668**	0.626	0.621	0.530	0.570
	*T*_*d*_×*T*_*p*_				
NR	0.160	0.151	**0.168**	0.100	0.080*
GR	0.156	0.156	**0.168**	0.042*	0.079*
IC	**0.233**	0.229	0.227	0.041*	0.198*
E	0.214	0.218	0.263*	0.016*	0.190*
Avg	0.191	0.189	**0.207**	0.050	0.137

**Table 3:** The drug-protein networks (DPN) used in the experimental evaluation are presented

*DPN*	|*drugs*|×|*proteins*|	|*Features*|	|*interactions*|
*NR*	54×26	54−26	90/1404 (6.4*%*)
*GR*	223×95	223−95	635/21185 (3*%*)
*IC*	210×204	210−204	1476/42840 (3.4*%*)
*E*	445×664	445−664	2926/295480 (1*%*)

We next evaluated BICTR by comparing it against state of the art DTI prediction approaches and the obtained AUROC and AUPR results are also presented in Tables 1 and 2, respectively. BICTR overall outperforms its competitors, affirming its effectiveness in DTI prediction. More specifically, BICTR surpasses BLMNII and STC in all prediction settings, both in terms of AUROC and AUPR. When it comes to NRLMF, BICTR yields better results in terms of AUROC in all settings and AUPR in *L*_*d*_×*T*_*p*_. The AUPR results obtained by BICTR are inferior in *T*_*d*_×*L*_*p*_ and *T*_*d*_×*T*_*p*_. Nevertheless, the differences are statistically significant only for the E dataset. In a case like that we could deduct that BICTR is better at maximizing true negatives (TN) while NRLMF is better at minimizing false positives (FP). In drug discovery the elimination of false positives, albeit important, is not as crucial as in other tasks because the possible hits or leads (i.e., positive interactions) will anyway get validated in the lab by (medicinal) chemists.

## Discussion

The obtained results indicate that output space reconstruction can elevate the performance of multi-output learning models, leading to more accurate DTI predictions. The effectiveness of BICTR was affirmed in all three DTI prediction settings. The contribution of the NRLMF-based step is substantial as it reconstructs the output space identifying potential non-reported drug-target interactions in the training set. This especially mitigates the problem of class imbalance. The performance improvement achieved by the output space reconstruction step was confirmed by conducted experiments, where BICTR clearly outperformed eBICT.

One could identify a connection between the approach presented in this chapter and the setting of Positive Unlabeled data (PU) learning [56]. Here, similar to PU learning, we acknowledge the lack of truly negative drug-target pairs. In the first step of our approach (matrix factorization-based) we reconstruct the interaction matrix of the networks, identifying the likely positive (interacting) drug-target pairs from the set of unlabeled ones (zeros in the interaction matrix). The subsequent supervised learning method is applied on a reconstructed interaction matrix, which consists of zeros (i.e., strong negative drug-target pairs), ones (i.e., interacting drug-target pairs), and fuzzy values (i.e., ambiguous drug-target pairs).

It should be also highlighted that the proposed method follows the inductive setup as the reconstruction of the output space takes place only in the training process. This means that after the training process is complete, one can perform predictions for new data (e.g., new candidate drugs). In addition, the employed matrix factorization step does not affect the interpretability of tree-ensemble learning which is subsequently introduced in the proposed DTI prediction method.

Furthermore, different from other approaches (e.g., NRLMF, STC, BLMNII), the proposed method does not require the training instances (feature vectors) to be kept, which can be vital for studies performed in large scale DTI networks. BICTR is not a similarity-based method and is perfectly applicable on other types of feature spaces. For example, one could use GO annotations or PFAM domains as protein related features and drug side effects or chemical compound interactions as drug-related features. Moreover, one could extract features from the network topology. In addition, as BICTR is a tree-ensemble method, it adopts all the advantages of decision tree based learning. It is scalable, computationally efficient, interpretable, and capable of handling missing values.

Moreover, synergistic learning approaches that employ multiple classifiers to yield predictions are not considered as competitors. BICTR can be clearly integrated into such mechanisms. The performance of BICTR can be also boosted by feature construction methods based on graph embeddings. Finally, we state that although matrix factorization (NRLMF) was employed for reconstructing the output space, other approaches could be used as well.

## Conclusion

In this paper we have presented a new drug-target interaction prediction approach based on multi-output prediction with output space reconstruction. We showed that multi-output learning models can manifest superior predictive performance when built on reconstructed networks. Tree-ensemble learning models and specifically ensembles of bi-clustering trees were deployed in this framework, constructing an accurate and efficient DTI prediction method. The proposed approach was compared against state of the art DTI prediciton methods on several benchmark datasets. The obtained results affirmed the merits of the proposed framework.

The learning method that was deployed here could be used to perform in silico predictions on large scale drug-target networks in the future. These predictions should get verified later in the lab, potentially revealing novel interactions.

## Method

In this section, we first discuss about the general structure of drug-target networks, present notations and describe different prediction settings. We then provide a broad description of tree-ensemble learning and multi-output prediction. Next, we present the individual mechanisms of bi-clustering trees and matrix factorization. Finally, the proposed DTI prediction approach is presented.

### Predicting drug-target interactions

Drug target interaction networks are heterogeneous networks, which are denoted as bi-partite graphs in graph theory. A DTI network consists of two finite sets of nodes *D*={*d*_1_,⋯,*d*_|*D*|_} and *P*={*p*_1_,⋯,*p*_|*P*|_}, that correspond to drugs and target proteins, respectively. Each node is represented by a feature vector. Drug-related features may consist of chemical structure similarities, drug side effects, or drug-drug interactions. Protein-related features may consist of protein sequence similarities, GO annotations, protein-protein interactions or protein functions. A link between two nodes of a DTI network corresponds to an existing interaction between the corresponding drug and target protein. The set of existing or not existing network links form an interaction matrix **Y**∈ℜ^|*D*|×|*P*|^. Every item *y*(*i*,*j*)∈**Y** is equal to 1 if an interaction between items *d*_*i*_ and *p*_*j*_ exists and 0 otherwise.

DTI prediction, a task also denoted as DTI network inference, can be handled as a supervised learning task and especially as a classification task on pairs of nodes. The goal is to build a model that receives a drug-target pair as input and outputs a probability that an interaction between these two pair nodes holds. In the most practical inductive setup, the learning model is built on a training set of drug-target pairs and after the learning process is complete, it can perform predictions for unseen pairs.

**Fig. 2:**
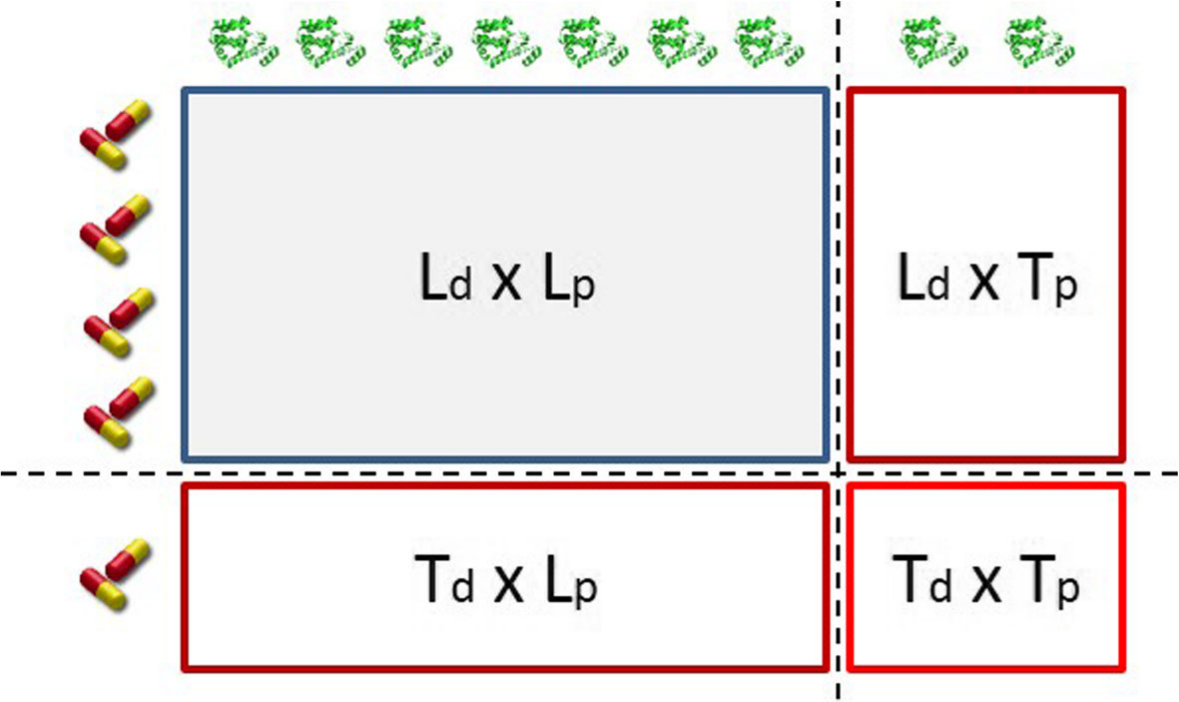
The prediction setting of a DTI network
- Learned drugs - Test targets (*L*_*d*_×*T*_*p*_): interactions between drugs that have been included in the learning procedure and new target proteins.
- Test drugs - Test targets (*T*_*d*_×*T*_*p*_): interactions between new drug candidates and new target proteins.

One can perform DTI predictions for new drugs, new target proteins, or new drug-target pairs. The latter is clearly more challenging. Predicting interactions between drugs and targets that are both included in the training set is considered a semi-supervised learning task and is not studied in this paper as we focus on supervised learning. The addressed prediction framework is demonstrated in Fig. 2. The (*L*_*d*_×*L*_*p*_) is the interaction matrix **Y**. DTI prediction tasks can be divided in 3 settings.

- Test drugs - Learned targets (*T*_*d*_×*L*_*p*_): interactions between new drug candidates and target proteins that have been included in the learning procedure.

The aforementioned prediction setting was thoroughly described in [26, 55, 57–59].

### Multi-output tree-ensembles

Decision tree induction algorithms [60] adopt a top-down architecture. The first node is called the root node. Every node is recursively split after applying a test to one of the instance features. A split quality criterion (e.g., entropy, variance reduction etc.) is employed to measure the quality of the split. The best split is selected and the tree growing process continues until the data contained in a node is pure w.r.t. the labels. The tree growing can also stop if a stopping criterion is reached. The last nodes of the tree are called leaves. Every leaf receives a label, which is typically the average or the majority of the labels of the containing instances. A new (unseen) instance will traverse the tree and end up in a leaf node. The label that corresponds to this leaf is then given as a prediction to the new instance.

Single trees often suffer from the overfitting effect and are considered as relatively unstable models. However, when they are extended to tree-ensembles [50], they often achieve state-of-the-art performance. The overfitting effect is also tackled by tree-ensembles. Several tree-ensemble approaches exist. Two of the most popular and effective ones are the random forests (RF) [50] and the extremely randomized trees (ERT) [61]. Typically, it is more challenging to interpret a tree-ensemble model than a single tree-based one. Nevertheless, there are strategies [62] that transform a tree-ensemble to a single tree, avoiding this way the loss of the interpretability advantage. Another advantage of tree-ensembles is their ability to rank the features, based on their contribution to the learning procedure. Although the predictive performance of tree-ensembles may slightly vary based on the different randomization seeds, they are considered as very stable predictors.

Moreover, most tree-based learning models can easily be applied to multi-output tasks, for example multi-label classification [63] or multi-target regression [14]. Multi-output models learn to predict multiple output variables simultaneously. In a DTI prediction task, the instances can be the drugs and the outputs (labels) are the drug-target interactions. When a new drug arrives, a set of labels is assigned to it. Each label of this set corresponds to an interaction between this drug and a target protein.

### Ensembles of bi-clustering trees

**Fig. 3:**
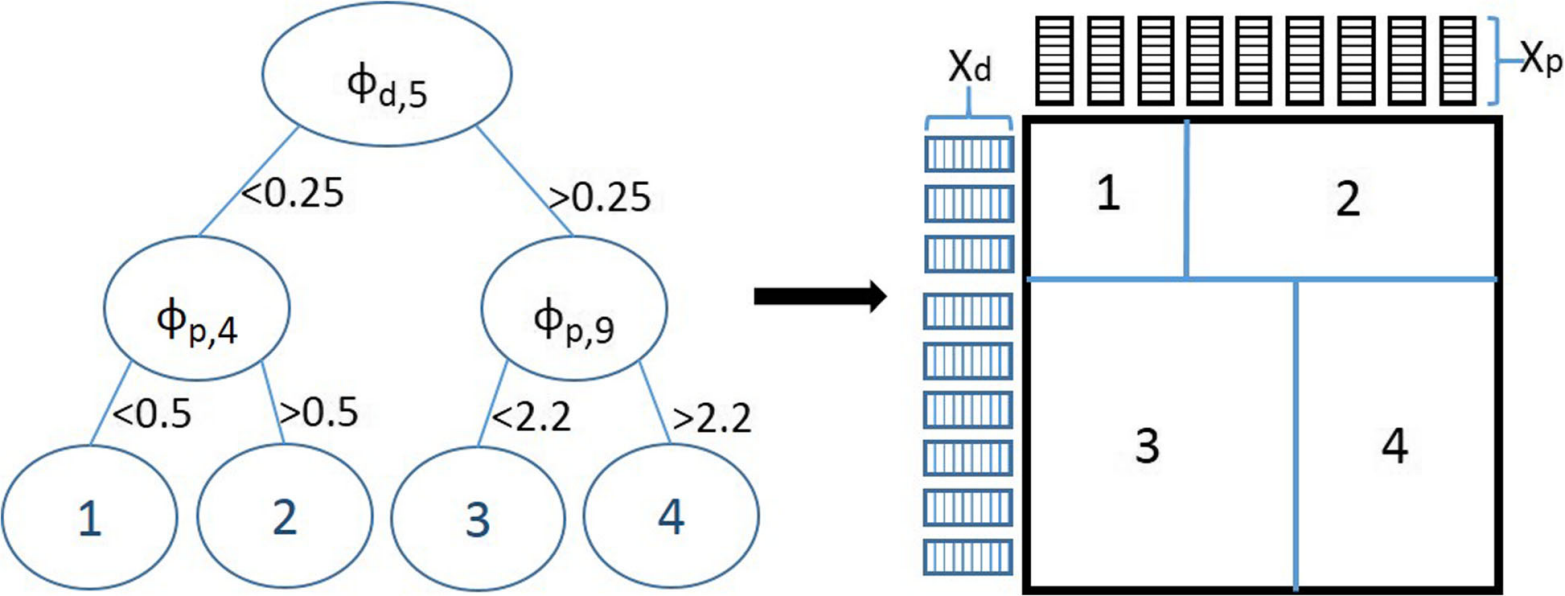
Illustration of a bi-clustering tree along with the corresponding interaction matrix that is partitioned by that tree. Let *ϕ*_*d*_ and *ϕ*_*p*_ be the features of the row and column instances, respectively

Pliakos et al. [27] proposed a *bi-clustering tree* for interaction prediction, extending a single multi-output decision tree to the global network setting. That tree model is shown in Fig. 3 [27]. The model is built on pairs of instances and predicts the interactions between them. This method was then extended to the tree-ensemble setting in [31], utilizing the ERT mechanism. The trees grow having a random sub-set of both row and column features as split candidates, inducing therefore a bi-clustering of the network. A split on a row feature corresponds to a row-wise partitioning of the matrix while a split on a column-feature to a column-wise one. The final predictions are generated as the average of the predictions yielded by each one of the trees that form the ensemble collection.

### NRLMF

In matrix factorization the goal is to compute two matrices that, when multiplied, approximate the input matrix. More concretely, in DTI prediction, the interaction matrix **Y**∈ℜ^|*D*|×|*P*|^ is used as input and the task is to compute two matrices, namely **U**∈ℜ^|*D*|×*k*^ and **V**∈ℜ^|*P*|×*k*^, so **UV**^*T*^≈**Y**. Matrices **U** and **V** are considered as *k*-dimensional latent representations of drugs and proteins, where *k*≪|*D*|,|*P*|.

The Neighborhood Regularized Logistic Matrix Factorization (NRLMF) [33] is principally based on LMF, modelling the probability $\hat y_{ij}$ that a drug *d*_*i*_ interacts with a target protein *p*_*j*_ as follows.

1 $$  \hat y_{ij} = \frac{\exp\left(\mathbf{u}_{i} \mathbf{v}_{j}^{T}\right)}{1 + \exp\left(\mathbf{u}_{i} \mathbf{v}_{j}^{T}\right)}  $$

The *k*-dimensional vectors **u**_*i*_ and **v**_*j*_ are latent representations of *d*_*i*_ and *p*_*j*_, respectively. The original LMF expression is extended with two regularization terms which contribute to avoid overfitting and two graph regularization terms that capture the drug corresponding and protein corresponding neighborhood information. More thoroughly, the two regularization terms that appear in the second line of Eq. (2) stem from the application of zero-mean Gaussian priors on the latent vectors of all drugs and targets. They prevent overfitting by favoring simple solutions that consist of relatively small values. The next two terms are graph regularization terms that contribute to the optimization procedure by learning the underlying manifolds in the data. The final objective function that is yielded is shown below:

2 $$ {}\begin{aligned} \min_{\mathbf{U},\mathbf{V}} & \sum_{i=1}^{|D|}\sum_{j=1}^{|P|}(1+cY_{ij}-Y_{ij})\ln{\left[1+\exp\left(u_{i}v_{j}^{T}\right)\right]}-cY_{ij}u_{i}v_{j}^{T} \\ & + \lambda_{d}||\mathbf{U}||_{F}^{2} + \lambda_{p}||\mathbf{V}||_{F}^{2} \\ & + \alpha \text{Tr}\left(\mathbf{U}^{T} \mathbf{L}^{d} \mathbf{U}\right) + \beta \text{Tr}\left(\mathbf{V}^{T} \mathbf{L}^{p} \mathbf{V}\right) \end{aligned}  $$

Parameters *λ*_*d*_,*λ*_*p*_,*α*, and *β* control the regularization terms while parameter *c* (*c*≥1) expresses the weight of observed interacting drug-target pairs to the optimization process. The idea was that these interacting pairs have been experimentally verified and are therefore more important than unknown pairs (i.e., *Y*_*ij*_=0). By adjusting *c*, we specify the importance level of interacting pairs to the optimization process. Moreover, when *c*>1 each interaction pair is treated as *c* positive pairs. This contributes to the mitigation of the class-imbalance problem.

### Bi-clustering trees with output space reconstruction

In our DTI task we assume that there are originally no truly negative drug-target pairs but only positive and unlabeled ones, which can be either positive (not reported yet) or negative. This setting is often referred to as Positive-Unlabeled (PU) learning setting [56]. The proposed approach learns bi-clustering trees with output space reconstruction (BICTR). This way tree-ensemble learning, a powerful supervised learning family of algorithms, is integrated with semi-supervised driven approaches, such as matrix factorization. Here, we promote ensembles of bi-clustering trees and NRLMF.

We first reconstruct the output space, exploiting neighborhood information, revealing underlying manifolds in the topology of the DTI network (i.e., interaction matrix) and alleviating class-imbalance. The input of our approach is the drug-related feature space *X*_*d*_, the target-related feature space *X*_*p*_, and the interaction matrix **Y**. We reconstruct the DTI network by learning matrices **U** and **V** based on Eq. 2. The new interaction matrix is denoted as $\hat {\mathbf {Y}}$ and every $\hat {y_{ij}} \in \hat {\mathbf {Y}}$ is computed as in Eq. 1. Although actually interacting pairs of the network have already received an increased level of importance through the reconstruction process, we support even further the verified interactions as follows:

3 $$  \hat {y}_{ij} = \left\{ \begin{array}{l l} 1, & \quad \text{if \ \(y_{ij} = 1\)}\\ \hat {y}_{ij}, & \quad \text{otherwise}. \end{array} \right.  $$

Next, we learn eBICT on the reconstructed target space. In more detail, the input for every tree in our ensemble is the drug-related feature space *X*_*d*_, the target-related feature space *X*_*p*_, and the reconstructed interaction matrix $\hat {\mathbf {Y}}$. The root node of every tree in our setting contains the whole interaction network and a partitioning of this network is conducted in every node. The tree growing process is based on both vertical and horizontal splits of the reconstructed interaction matrix $\hat {\mathbf {Y}}$. The variance reduction is computed as $Var=\sum _{j}^{|P|} Var\left (\hat {\mathbf {Y}}_{j}\right)$ when the split test is on *ϕ*_*d*_∈*X*_*d*_ and $Var=\sum _{i}^{|D|} Var\left (\hat {\mathbf {Y}}^{T}_{i}\right)$ when the split test is on a *ϕ*_*p*_∈*X*_*p*_.

The NRLMF-based target space reconstruction step of the proposed DTI prediction strategy boosts the predictive performance of the eBICT while preserving all the advantages of tree-ensembles, such as scalability, computational efficiency, and interpretability. An analysis of the computational efficiency and interpretability of bi-clustering trees took place in [27]. The approach that is proposed here, despite being integrated with matrix factorization, continues to follow the inductive setup. In more detail, the output space reconstruction process takes place only in the training process. After the training model is complete, new instances that may arrive (e.g., new candidate drugs) just traverse the grown bi-clustering trees and predictions are assigned to them based on the leaves in which they end up.

## Data

We employed 4 benchmark datasets that represent drug-target interaction networks [64]. The characteristics of each network are shown in Table 3. More specifically, this table contains the number of drugs, proteins, and existing interactions in every network. The number of features used to represent each sample (drug or protein) is also displayed.

The datasets in [64] correspond to 4 drug-target interaction networks where the interactions between drugs and target proteins are represented as binary values. In these networks, compounds interact with proteins that belong to 4 pharmaceutically useful categories: nuclear receptors (NR), G-protein-coupled receptors (GR), ion channels (IC), and enzymes (E). The features that describe the drugs are similarities based on their chemical structure. The features representing the target proteins correspond to similarities based on the alignment of protein sequences. The sequence similarities were calculated according to the normalized Smith-Waterman score.

## Data Availability

The data and materials used in this study can be found here: http://www.montefiore.ulg.ac.be/~schrynemackers/datasets, http://web.kuicr.kyoto-u.ac.jp/supp/yoshi/drugtarget/, https://github.com/gerdinard/myprojects/

## Abbreviations

AUPR: Area under precision recall curve
AUROC: Area under the receiver operating characteristic curve
BICTR: BI-Clustering Trees with output space Reconstruction
DPI: Drug-protein interaction
DTI: drug-target interaction
E: Enzymes
eBICT: Ensemble of bi-clustering trees
ERT: Extremely randomized trees
GR: G-protein-coupled receptors
IC: Ion channels
LMF: Logistic matrix factorization
MLkNN: Multi-label k-nearest neighbor
NR: Nuclear receptors
NRLMF: Neighborhood regularized logistic matrix factorization
RF: Random forests
STC: Super target clustering
